# Virulence factor landscape of a *Staphylococcus aureus* sequence type 45 strain, MCRF184

**DOI:** 10.1186/s12864-018-5394-2

**Published:** 2019-02-08

**Authors:** Vijay Aswani, Fares Najar, Madhulatha Pantrangi, Bob Mau, William R. Schwan, Sanjay K. Shukla

**Affiliations:** 1 Department of Internal Medicine & Pediatrics, University at Buffalo, Buffalo, New York USA; 2 Department of Chemistry & Biochemistry, University of Oklahoma, Norman, OK USA; 3 Center for Human Genetics, 1000 North Oak Avenue # MLR, Marshfield, WI 54449 USA; 4 Wisconsin Institute for Discovery, University of Wisconsin, Madison, WI USA; 5 Department of Microbiology, University of Wisconsin –La Crosse, La Crosse, WI USA

**Keywords:** *Staphylococcus aureus*, Virulence factors, Necrotizing fasciitis, ST45, enterotoxin gene cluster

## Abstract

**Background:**

We describe the virulence factors of a methicillin-sensitive *Staphylococcus aureus* sequence type (ST) 45 strain, MCRF184, (*spa* type t917), that caused severe necrotizing fasciitis in a 72-year-old diabetic male. The genome of MCRF184 possesses three genomic islands: a relatively large type III *ν*Saα with 42 open reading frames (ORFs) that includes superantigen- and lipoprotein-like genes, a truncated *ν*Saβ that consists mostly of the enterotoxin gene cluster (*egc*), and a *ν*Saγ island with 18 ORFs including α-toxin. Additionally, the genome has two phage-related regions: phage φSa3 with three genes of the immune evasion cluster (IEC), and an incomplete phage that is distinct from other *S. aureus* phages. Finally, the region between *orfX* and *orfY* harbors a putative efflux pump, acetyltransferase, regulators, and mobilization genes instead of genes of SCC*mec*.

**Results:**

Virulence factors included phenol soluble modulins (PSMs) α1 through α4 and PSMs β1 and β2**.** Ten ORFs identified in MCRF184 had not been reported in previously sequenced *S. aureus* strains.

**Conclusion:**

The dire clinical outcome in the patient and the described virulence factors all suggest that MCRF184, a ST45 strain is a highly virulent strain of *S. aureus*.

**Electronic supplementary material:**

The online version of this article (10.1186/s12864-018-5394-2) contains supplementary material, which is available to authorized users.

## Background

The ability of *S. aureus* to colonize and infect humans comes from a large arsenal of virulence genes including genes for proteins to attach to host tissue, tissue-degrading enzymes, leukocidins, antibiotic-resistance, pyrogenic toxins, and immunomodulating proteins [1]. A number of *S. aureus* genomes have been sequenced to identify potential new virulence genes or novel combinations of known virulence genes [2]. These studies have led to the identification of new genomic islands and genetic elements, which harbor known and putative toxins, phenol-soluble modulins, and accessory genes to virulence [3–6]. Differences in virulence of *S. aureus* strains, however, may be due to even small differences in genome sequence: Kennedy et al [7] studied genetic variation in USA300 MRSA strains and found that large differences in virulence in a mouse sepsis model occurred among strains with relatively few genetic differences. Single SNP differences have recently been demonstrated to underpin the virulence of some strains [8, 9]. Similarly, the insertion of IS256 (a transposable element) into the promotor of the *rot* gene increased virulence [10]. Panton-Valentine leukocidin (PVL), a major virulence factor of *S. aureus* has been shown to have a direct role in necrotizing fasciitis [5]. We describe here the virulence traits of MCRF184, a methicillin-sensitive, ST45 strain that caused a debilitating necrotizing fasciitis in a diabetic man, necessitating the amputation of the patient’s leg to save his life.

## Results

### Overview of antimicrobial resistance and virulence gene content

MCRF184 is a methicillin-susceptible strain that belongs to sequence type (ST) 45 and *spa* type t917. This strain was recovered during both the early and late stages of the infection of the leg [11]. Among some of the known virulence factors of *S. aureus*, the genome of this strain harbored clumping factors genes *clfA* and *clfB*, fibronectin binding protein gene *fnbA* but not *fnbB*, collagen binding adhesion gene *cna*, intracellular adhesion gene *icaA* and newly identified toxin genes – *bsa,* staphylococcal superantigen-like gene 1 (*ssl1*), and *lpl110* (Table 1). The staphylococcal enterotoxins, staphylococcal superantigen-like (*ssl*) genes, and genes involved in immune evasion were present on mobile genetic elements. MCRF184 was negative for toxic shock syndrome toxin (*tst*), and the Panton-Valentine leucocidin (*lukSF*-*PV*).

**Table 1:** Major virulence-related genes in *S. aureus* strain, MCRF184

	Locus	Location
Enterotoxins	CKU_1443 SE	core
	CKU_1636 *seg*	*v*Saβ
	CKU_1637 *sen*	*v*Saβ
	CKU_1638 *seu*	*v*Saβ
	CKU_1639 *sei*	*v*Saβ
	CKU_1640 *sem*	*v*Saβ
	CKU_1641 *seo*	*v*Saβ
Exotoxins	CKU_0360 *ssl1*	*v*Saα
	CKU_0361 *ssl2*	*v*Saα
	CKU_0362 *ssl4*	*v*Saα
	CKU_0363 *ssl3*	*v*Saα
	CKU_0365 *ssl5*	*v*Saα
	CKU_0366 *ssl9*	*v*Saα
	CKU_0367 *ssl10*	*v*Saα
	CKU_0370 *ssl11*	*v*Saα
	CKU_0998 *ssl12*	*v*Saγ
	CKU_0999 *ssl13*	*v*Saγ
	CKU_1000 *ssl14*	*v*Saγ
Exfoliative toxin	CKU_1005 *eta*	*v*Saγ
Alpha-hemolysin	CKU_0995 *hla*	*v*Saγ
Beta-hemolysin	CKU_1753 *hlb*	*ф*Sa3
Delta-hemolysin (RNAIII)	CKU_2494 *hld*	core
Gamma-hemolysin Component	CKU_2175 *hlgA*	core
Gamma-hemolysin Component	CKU_2176 *hlgC*	core
Gamma-hemolysin Component	CKU_2177 *hlgB*	core
Adhesins		
Collagen-binding protein	CKU_2442 *cna*	core
Fibronectin-adhesin	CKU_2253 *fnbA*	core
Elastin adhesin	CKU_1327 *ebpS*	core
Laminin-adhesin	CKU_0713 *eno*	core
Fibrinogen	CKU_0723 *clfA*	core
Fibrinogen	CKU_2384 *clfB*	core
Fibrinogen	CKU_0989 *fib*	core
Fibrinogen	CKU_0496 *sdrC*	core
Exoenzymes		
Serine protease	CKU_0857 *htrA*	core
Serine V8 protease	CKU_0881 *sspA*	core
Cysteine protease	CKU_0880 sspB	core
Cysteine protease	CKU_0879 sspC	core
Lipase precursor	CKU_0273 geh	core
Lipase precursor	CKU_2426 *gehC*	core
Lipase	CKU_0588 *lipA*	core
Esterase	CKU_2106	core
Hyaluronate lyase	CKU_1961 hysA2	core
Termonuclease	CKU_1173 *nucH*	core
Cell wall hydrolase	CKU_1081 *lytN*	core
Zinc metalloprotease	CKU_1096	core
Clp protease proteolytic subunit	CKU_0704 *clpP*	core
Clp protease ATP binding subunit	CKU_0813 *clpB*	core
Clp protease ATP binding subunit	CKU_1509 *clpX*	core
Clp protease ATP binding subunit	CKU_2300 *clpL*	core
Phenol Soluble Modulins		
PSMα1	426,966 to 426,902	core
PSMα2	426,870 to 426,805	core
PSMα3	426,752 to 426,685	core
PSMα4	426,620 to 426,559	core
PSMβ1	CKU_1007	core
PSMβ2	CKU_1008	core
PSMδ	1,955,755 to 1,955,676	core
Immunomodulators		
Staphylokinase	CKU_1760 *sak*	*ф* Sa3
Chemotaxis inhibiting protein	CKU_1758 *chp*	*ф* Sa3
Complement inhibitor	CKU_1757	*ф* Sa3
Immunoglobulin G binding protein A	CKU_0065 *spa*	core
Immunoglobulin G binding protein	CKU_2174 *sbi*	core
Lipoprotein like gene products	CKU_2474 *lpl1*	*v*Saα
Lipoprotein like gene products	CKU_0373 *lpl2*	*v*Saα
Lipoprotein like gene products	CKU_0374 *lpl3*	*v*Saα
Lipoprotein like gene products	CKU_0375 *lpl4*	*v*Saα
Lipoprotein like gene products	CKU_0376 *lpl5*	*v*Saα
Lipoprotein like gene products	CKU_0377 *lpl6*	*v*Saα
Lipoprotein like gene products	CKU_0378 *lpl*	*v*Saα
Virulence related genes		
Biofilm genes	CKU_2420 *icaR*	core
	CKU_2421 *icaA*	core
	CKU_2422 *icaD*	core
	CKU_2423 *icaB*	core
	CKU_2424 *icaC*	core
Leukocidin GH	CKU_1786 *lukGH*	core
Regulatory genes		
*S. aureus* exotoxin (SaeRS)	CKU_0640 *saeS*	core
	CKU_0641 *saeR*	core
Staphylococcal accessory regulator (sarA)	CKU_0551 *sarA*	core
Sigma factor B	CKU_1825 *sigB*	core
Repressor of Toxins	CKU_1594 *rot*	core

### Mobile genetic elements

**Fig. 1:**
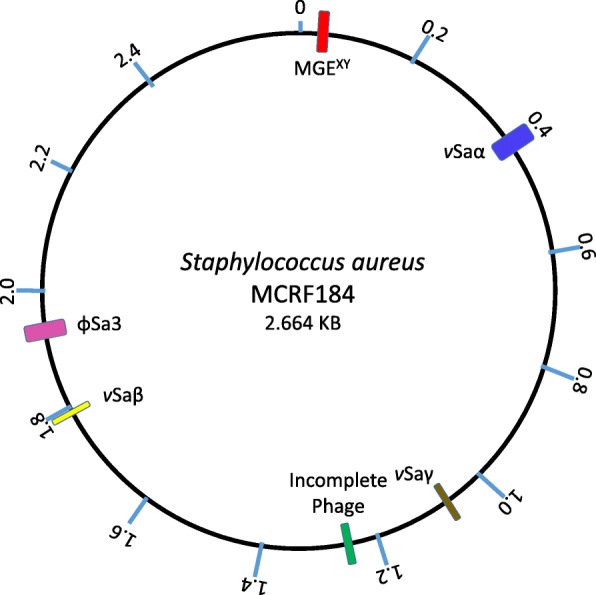
Circular representation of the MCRF184 genome. Virulence genomic islands are marked: *v*Saα [blue], *v*Saβ [yellow], *v*Saγ [brown], Incomplete phage [green], φSa3 [pink] and MG^XY^ [red]

The MCRF184 strain harbored six MGEs (Fig. 1): *v*Saα, *v*Saβ, *v*Saγ, φSa3, an incomplete phage, and a newly identified region between *orfX* and *orfY*, named MGE^*XY*^ that also harbored mobilization genes. The incomplete phage has not been previously described, and the MGE^*XY*^ harbored novel combinations of sequences. The MCRF184 genome did not include intact pathogenicity islands [12], plasmids, or integrative conjugative elements (ICE*6013*, Tn*916*/Tn*5801*) [13].

### Genomic islands of MCRF184

#### vSaα

**Fig. 2:**
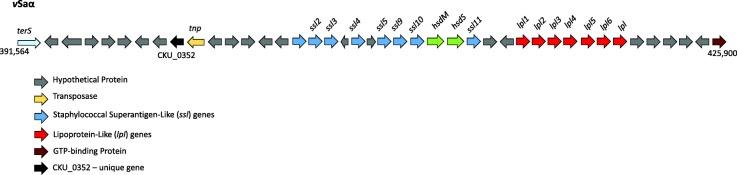
*v*Saα genomic island showing lipoprotein-like and staphylococcal superantigen like genes along with mobile genetic elements

Genomic islands, generally 10 to 200 kb long, are a cluster of genes acquired by horizontal transfer [14]. The *v*Saα was a type III genomic island (Fig. 2) and harbored alleles of eight *ssl* and seven lipoprotein-like (*lpl*) genes. The *v*Saα region was nearly identical to *v*Saα of two other ST45 strains, CA-347 [15] and an unpublished genome, CFSAN007835 (GenBank # CP017685.1). In *v*Saα, eleven SNPs accounted for the differences between MCRF184 and CA347 (Table 2), eight of which were in protein coding regions—five of which would result in amino acid substitutions and one in a truncated protein in both MCRF184 and CA347. All but two of these changes in coding regions were in hypothetical proteins; of the two other changes, one was in an exotoxin gene and the other in the host specificity gene, *hsdS (*CKU_0369) of the restriction modification system.

**Table 2:** Comparison of *v*Saα of MCRF184 with the strain, CA347 identifying SNP differences between them

Nucleotide position	AA Change	Region	CDS	CDS Position	Change	Codon Change	Polymorphism Type	Protein Effect
392,566	A - > T	HP	CKU_2476	292	C - > T	GCT - > ACT	SNP (transition)	Substitution
395,313		non-coding			A - > C		SNP (transversion)	n/a
398,723		HP	CKU_0352	161	A - > T		SNP (transversion)	Truncation
399,587	A - > T	HP	CKU_0355	52	C - > T	GCA - > ACA	SNP (transition)	Substitution
401,239		non-coding			T - > C		SNP (transition)	n/a
401,244		non-coding			A - > G		SNP (transition)	n/a
404,499	I - > K	exotoxin	CKU_0361	473	T - > A	ATA - > AAA	SNP (transversion)	Substitution
412,382	I - > V	hsdS	CKU_0369	76	A - > G	ATT - > GTT	SNP (transition)	Substitution
418,429	G - > W	HP	CKU_0374	658	G - > T	GGG - > TGG	SNP (transversion)	Substitution
423,848		HP	CKU_0381	168	C - > T	GGC - > GGT	SNP (transition)	None
423,933	F - > V	HP	CKU_0381	253	T - > G	TTT - > GTT	SNP (transversion)	Substitution

#### vSaβ

**Fig. 3:**
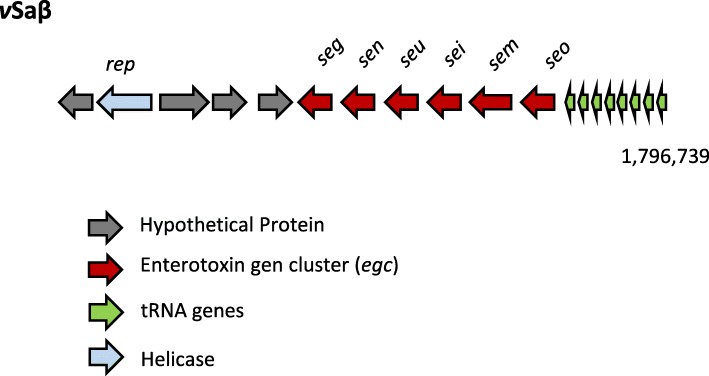
*v*Saβ genomic island showing genes of the enterotoxin gene cluster (*egc*)

The *v*Saβ of MCRF184 was truncated compared to *v*Saβ in MW2 and USA300FPR3757. It harbored eleven ORFs including the enterotoxin gene cluster (*egc*) genes: *seg*, *sen*, *seu sei*, *sem* and *seo* (Fig. 3), and was nearly identical in genes present in all three ST45 strains. Four genetic differences were noted in the *v*Saβ islands between MCRF184 and CA347 strain (Table 3), three of which were single nucleotide polymorphisms (SNPs). A significant additional difference was the deletion of two transposases in MCRF184, but present in CA347 strain. Furthermore, one of the SNPs in *sen* would lead to a truncated protein in MCRF184. The region containing two genes – a *rep* gene coding for a helicase and a second gene coding for a hypothetical protein, between positions 1,785,972 and 1787, 688 were unique to the three CC45 strains and not found in other *S. aureus v*Saβ islands. The observation that a hypothetical protein and the helicase were found in the three ST45 strains but absent from the other *v*Saβ islands sequenced could be of significance for the ST45 strains’ pathogenicity.

**Table 3:** Comparison of *v*Saβ of MCRF184 with strain, CA347 identifying SNP differences between them

Nucleotide Position	AA Change	Region	CDS	CDS Position	Change	Codon Change	Polymorphism Type	Protein Effect
1787,882		CDS			1223 bases	n/a	deletion	loss of two transposases, CA347_RS09315 and CA347_RS09320
1,789,959		noncoding			C - > T		SNP (transition)	
1,791,116	M- > STOP	*sen*	CKU_1637	756	T - > A		SNP (transversion)	truncation
1,795,127	N - > D	*seo*	CKU_1641	235	T - > C	AAT - > GAT	SNP (transition)	Substitution

The genomic islands, *v*Saα and *v*Saβ generally exist in four allelic forms in *S. aureus* strains and their specificity is determined by the structural differences in *hsdS* (host specificity determinant), a rapidly evolving gene with amino acid sequence level identity across the *S. aureus* genomes of less than 66% [3]. *v*Saβ lacked the *hsdS* (Fig. 3).

#### vSaγ

Comparison of the *v*Saγ sequence between the two other ST45 strains, MCRF184 and CA-347, revealed conserved gene order and no amino acid differences. Comparing nucleotide and amino acid sequences between them (Table 4), there were only three differences in protein-coding regions, none of which resulted in an amino acid change. A comparison with other ST types *S. aureus* strains showed conservation of gene composition.

**Table 4:** Comparison of *v*Saγ of MCRF184 with strain, CA347 identifying SNP differences between them

Nucleotide Position	AA Change	Region	CDS	CDS Position	Change	Codon Change	Polymorphism Type	Protein Effect
1,086,484	No	XTP/dITP diphosphatase	CKU_0983	87	T > C	TAT > TAC	SNP (transition)	None
1,071,553	A	HP	CKU_0988	1	A > T	ATG > TAG	SNP (transversion)	None
1,077,630	C	*ssl12*	CKU_0998	145	C > G	ACA > AGA	SNP (transversion)	None

**Fig. 4:**
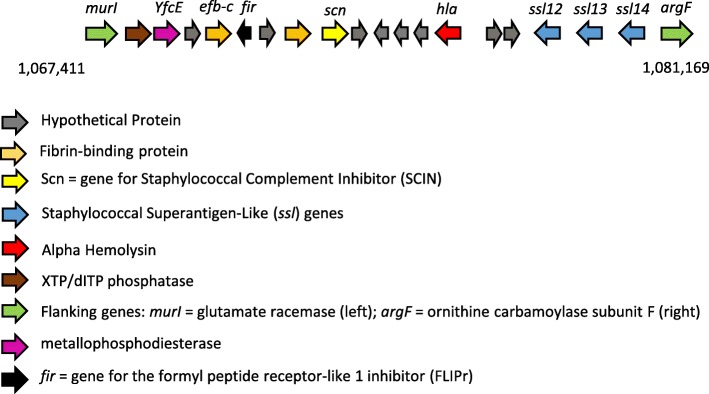
*v*Saγ genomic island. Included in this island is the IEC2 group of genes

This genomic island contains the IEC2 cluster, including the α-haemolysin (Hla) and the prototype βPFT of *S. aureus*. The *v*Saγ (Fig. 4) was flanked by the genes *murI* (glutamic racemase) and *argF* (ornithine transcarbamoylase subunit F). It additionally contained three more *ssl*s.

### The phages of MCRF184

#### φSa3

**Fig. 5:**
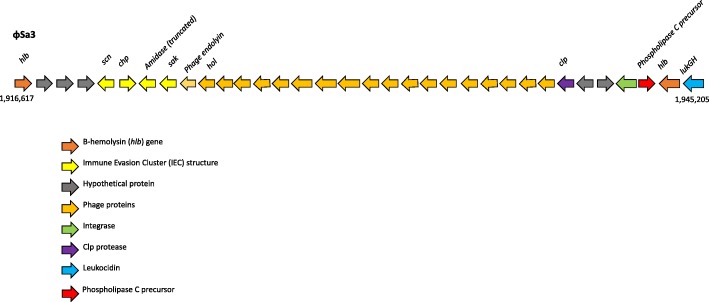
φSa3 phage-derived region showing genes of the immune evasion cluster (IEC)

The φSa3 (Fig. 5) was inserted into the *hlb* gene, making it a β-hemolysin-converting bacteriophage (βC-φ). This phage is known to carry IEC1, which is variable in gene content among strains [16]. In MCRF184, IEC1 consists of *sak – (truncated amidase) – chp – scn* suggesting that it is an IEC type B [17]. The truncated amidase is not unique to MCRF184, and an intact amidase is upstream of *sak* and forms part of the endolysin-holin lytic module of the phage. There was also *lukGH* genes located downstream of the phage element (Fig. 5), representing a core genome virulence factor in MCRF184.

#### An incomplete phage

**Fig. 6:**
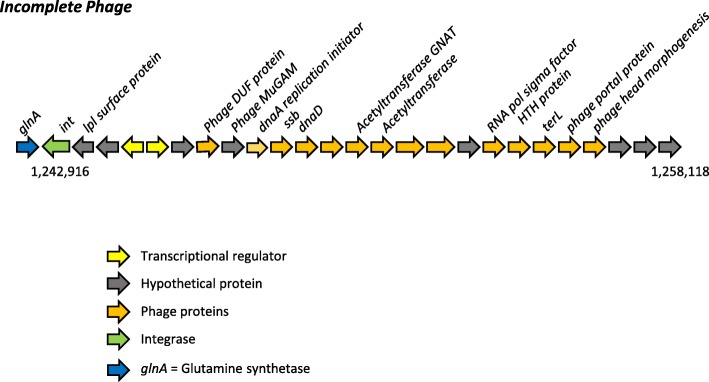
Incomplete phage showing phage-derived genes

A novel incomplete phage was located between nucleotide positions 1,242,209 to 1,258,118 (Fig. 6). PHASTER analysis found it to be an incomplete prophage (PHASTER score 40; < 70 considered incomplete). Twenty of the 27 proteins were identified as phage proteins. Three of the 27 proteins matched staphylococcal phage φNM3. The complete sequence of this incomplete phage had a > 99% sequence identity with ST45 strains CA-347 and CFSAN007835. The gene content was unusual in having a terminase large subunit gene (*terL*) instead of a small subunit gene (*terS*), and in having a phage head morphogenesis gene. Interestingly, SaPIbov5 is known to have *terL* but not *terS*, and is mobilized by both *pac*- and *cos*-type helper phages [16]. The glutamine synthetase gene is not known to be used as an integration site by SaPIs, but it is used by an unrelated 30 kb phage, ϕ909 described in *S. epidermidis* [18]. The integrase of this incomplete phage was distinct from the groups defined for *S. aureus* phage [19] and SaPIs [20].

#### MGE^XY^

**Fig. 7:**
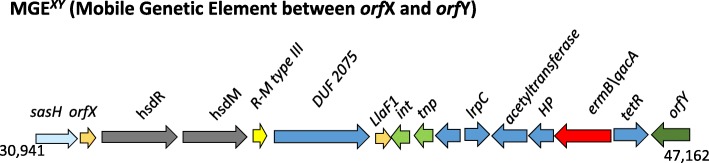
MGE^*XY*^ (Mobile Genetic Element between *orf*X and *orf*Y) is a region on the genome showing unique mix of antibiotic-resistance genes and mobile genetic elements. Other *S. aureus* genome regions are shown for comparison. *orfX* is also known as *rlmH*

The SCC*mec* cassette in MRSA is usually present at the 3′ end of the conserved gene *orfX,* an rRNA methyltransferase at a position ~ 34,000 base pairs from the origin of the replication [21]. The region between *orfX* and *orfY*, a tRNA dihydrouridine synthetase is known to be highly variable in gene content among *S. aureus* strains [22, 23]. In MCRF184, this region has a series of restriction-modification genes (*hsdR*, *hsdM*, and R-M type III) and a unique combination of putative antimicrobial resistance genes (*emrB/qacA, tetR*) located near the mobilization genes, *int* and *tnp* for transposon and integrase (Fig. 7). The putative efflux pump, *emrB/qacA*, is among those known for *S. aureus* [24]. The position of the *hsdR* and *hsdM* genes and the R-M type III system in this location of the *S. aureus* genome appears to be well conserved (Fig. 7). However, the presence of *emrB/qacA, tetR* and *int* and *tnp* in this region appear unique to MCRF184, CA-347, CFSAN007835 (all ST45 types) and an ST508 *S. aureus* isolated from a Buruli ulcer [25].

### The phenol soluble modulins of MCRF184

Phenol soluble modulins (PSMs) are a family of amphipathic, alpha-helical peptides that have multiple roles in pathogenesis and are critical determinants of staphylococcal virulence [26]. In MCRF184, we identified all four α peptides, the two β peptides and the δ-peptide (Additional file 1: Figure S1A and B). We further confirmed the PSMs by determining their predicted structures: characteristic α-helical secondary structures that were amphipathic—hydrophilic on one side and hydrophobic on the other—using PEP-FOLD [27, 28] for the alpha PSMs and SWISS-MODEL Workspace [29] for the beta PSMs (Additional file 1: Figure S1C).

## Discussion

The whole genome sequence analysis of MCRF184, a clinically virulent and aggressive strain showed virulence features in common with two other ST45 strains, CA-347 and CFSAN007835 available in GenBank. However, these virulence features of the other genomes have not been described. Our analysis of the genomic islands of MCRF184 points to several distinctive virulence features: a streamlined νSaβ that mostly consists of the *egc*, and an MGE^XY^ that appears to be unique to ST45 strains of *S. aureus*.

With regards to the virulence factors of the νSaα, Nguyen [30] showed that deletion of the *lpl* cluster, which is also present in the νSaα genomic island of MCRF184, prevents the stimulation of the production of proinflammatory cytokines in human monocytes, macrophages, and keratinocytes. They further demonstrated that purified lipoprotein, Lpl1 was able to elicit a TLR2-dependent response and that heterologous expression enhanced their immune stimulatory activity, particularly contributing to the invasion of *S. aureus* into human keratinocytes and mouse skin, compared to cells without these virulence genes. Thus, the *lpl* cluster of MCRF184 *v*Saα may help stimulate virulence by stimulating a host inflammatory response that can cause symptoms of pain, swelling, erythema and fever.

The *egc* in MCRF184 encodes six genes, which belong to a superantigen family that are capable of triggering a massive toxic shock response [31]. Proteins encoded by *egc* are not reported to be highly immunogenic, but they can evade immune response due to lack of neutralization by the human sera [32]. In a comprehensive study done by Roetzer et al [33], it has been shown that 1) supernatants from a strain harboring *egc* were sufficient for a lethal outcome in rabbits, 2) different quantities of *egc* encoded enterotoxins are produced by *S. aureus* isolates, 3) 10 nanograms of expressed and purified recombinant SEI and SEN was lethal at 24 h and 48 h, and 4) *sei* and *sen* appear to play a more important role in virulence compared to the other *egc* genes. Stach et al [34], in a rabbit model of infective endocarditis, investigated the role of *tstH* and individual genes of *egc* and in a USA200 genetic background and noted that proteins from both genes independently contributed to development of vegetation and infective endocarditis. Proteins made by *sem*, *seo,* and *seu* contributed to the vegetation formation, and deletions of *tstH* and *egc* decreased the vegetation size. Furthermore, Johler et al [35] reported outbreaks of staphylococcal food poisoning and emetic activity from *egc*-harboring *S. aureus* belonging to clonal complex CC9 and CC45. These observations suggest that even though MCRF184 had a truncated *v*Saβ island, the virulence imparted by the *egc* genes alone could account for significant virulence through their modulation of the immune system, particularly in the 72-year-old diabetic male with the life and limb-threating necrotizing fasciitis. Furthermore, the presence of an IEC in φSa3 could have contributed to evading phagocytosis of the pathogen. Another interesting aspect of the MCRF184 genome is that it had three ferrichrome-binding proteins—*fhuA*, *fhuB,* and *fhuD—*important for growth under iron-restricted conditions [25]. The MCRF184 φSa3 was integrated into the *hlb* and extended to position 2 genes upstream of *groEL*.

The MGE^*XY*^ region of MCRF184 was identical to the ones found in CA-347 and the *S. aureus* Buruli ulcer isolate [25]. The region encodes a restriction-modification system (*hsdR*/*hsdM*) and included an *ermB*/*qacA* drug resistance transporter gene of the major facilitator superfamily (MFS) including an integrase, a transposase, a *tetR*/*acR* family transcriptional regular [36], and a flanking tRNA. The presence of all three types of PSMs—four α-types, two β-types and one δ-type—and their ability to enhance virulence through cytolysis of cells of the immune system and biofilm formation suggest further mechanisms for the enhanced virulence of MCRF184.

Wang et al [6] showed that psmα mutants were severely attenuated in their ability to cause subcutaneous abscesses in the skin of mice compared with the wild-type strain. Thus, the psmα toxins in MCRF184 could have contributed significantly to their virulence in causing necrotizing fasciitis and in their ability to cause soft tissue infections in a mouse model studied. PSMs in *S. aureus* contribute to the formation of biofilms and detachment of biofilm clusters for dissemination. The presence of the PSMs in MCRF184 and the biofilm genes (Additional file 1: Figure S1), CKU_2420 through CKD_ 2424 may again enhance the necrotizing fasciitis capability of the strain. PSMs of the α-type are known to be cytolytic, and the δ-toxin has been shown to lead to mast cell degranulation. The δ-toxin of MCRF184 (Table 1) is found within the RNAIII gene (CKU_2494) downstream of the *agrB* gene (CKU_1795). The RNAIII is the effector of the Agr system [6]. An interesting role for the PSMα3 of MCRF184 is their formation of amyloids [37] that are cross-α-fibrils, a newly discovered mode of self-assembly characterized by the piling of α-helices (Additional file 1: Figure S1C) perpendicular to the fibril axis. Similarly, PSMα1 promotes biofilm stability by preventing disassembly by matrix degrading enzymes and mechanical stress [38].

## Conclusion

MCRF184’s genome contained several distinguishing features, such as a truncated *v*Saβ, an incomplete phage and a MGE^*XY*^ not seen other *S. aureus* STs. Virulence of this strain likely came from its unique genetic background and SNPs in regulatory elements of virulence genes including *egc*. It also highlights the fact that there are highly virulent *S. aureus* strains out there which despite lacking the known potent toxins such as Panton-Valentine leukocidin, alpha toxin, etc., are still capable of causing serious, debilitating disease in susceptible individuals.

## Methods

The study was approved by the Marshfield Clinic Research Institute’s Institutional Review Board under the study number SHU10105 with waiver of documentation of informed consent.

### Bacterial strain

The *S. aureus* strain MCRF184, was isolated multiple times from a 72-year-old male during the treatment of his necrotizing fasciitis [11], and we sequenced the first isolate’s genome.

### Genome sequence and comparative analysis

The MCRF184 genome was sequenced by both a shotgun (single end) and a paired end libraries on a Roche 454 and assembled and annotated as described in Aswani et al 2016 [39] (BioProject PRJNA39571, BioSample SAMN02953006, GenBank CP014791). Its multilocus sequence type (MLST) and lack of SCC*mec* was deduced from the genome sequence and confirmed by Sanger sequencing and PCR.

### Identification of genomic islands and other putative virulence genes

Genomic islands in the MCRF815 genome were confirmed using IslandFinder [40] and Zisland explorer [41]. Virulence factors were further identified using VirulenceFinder [42].

### Prophage analysis

PHASTER (PHAge Search Tool - Enhanced Release) was used to analyze prophages in the genome [43]. This program is based on an earlier version called PHAST that detects prophage regions by examination prophage genes and their distance from each other [44].

### Single nucleotide polymorphism (SNP) analysis

SNP Analysis was performed with Geneious 11.0.3 (https://www.geneious.com). To perform the analysis, DNA sequences of the three genomic island, *v*Saα, *v*Saβ, and *v*Saγ from MCRF184 were aligned with the corresponding island sequences of CA-347 using Geneious Alignment, a global alignment with free end gaps with a 65% similarity (5.0/− 4.0) cost matrix and gap open penalty of 12 and gap extension penalty of 3. Once aligned, Geneious called variants/SNPs and reported effect of the variants on protein translation using a Bacterial Genetic Code, and merging adjacent variations.

### PSM peptide structure modelling

The predicted protein structure of the α-PSMs were determined using SWISS_MODEL Workplace [29] (https://swissmodel.expasy.org/interactive). The SWISS_MODEL accepted the peptide sequence as input, with no additional parameters required and it generated a PDB file formatted secondary structure, and a descriptive report. The protein structure of the β-PSMs was modelled using PEP-FOLD3 [27, 28] (http://mobyle.rpbs.univ-paris-diderot.fr/cgi-bin/portal.py#forms::PEP-FOLD3). The input was the PSM amino acid sequences to generate a 3-D structure of the peptide using sOPEP (structure Optimized Potential for Efficient structure Prediction) as the model sorter after 100 independent simulations.

## Additional file

Additional file 1: **Figure S1.** Phenol soluble modulins (PSM) of *S. aureus* MCRF184. Panel 1A shows the amino acid sequences of the alpha and beta PSMs arranged from the N-terminus to the C-terminus. Numbers at the right show the net charge of the peptides at pH 7.0, rounded to whole numbers, and considering N-formylation of the initial methionine residue. The highlighted text identifies the amphipathic α-helical domain. Panel 1B shows the location of the genes coding for these PSMs in the genome of MCRF184 core genome. Panel 1C shows the predicted structure of the PSMS using PEP-FOLD (for the alpha PSMs) and SWISS-MODEL Workspace (for the beta PSMs). The residues are color-coded by their position in the peptide chain. Each chain is drawn as a smooth spectrum from blue through green, yellow and orange to red. The N-terminus of the peptides is colored red and the C terminuses are drawn in blue. The structures show the characteristic α-helical structure of the C-terminus ends of the PSMs. (PDF 334 kb)

## Abbreviations

ICE: integrative conjugative elements
IEC: immune evasion cluster
*lpl*: lipoprotein-like
MGE: mobile genetic element
ORFs: open reading frames
PSMs: phenol soluble modulins
PVL: Panton-Valentine leukocidin
SNP: single nucleotide polymorphisms
*Ssl*: staphylococcal superantigen-like
ST: sequence type
