# Supplementary material for: Virulence factor landscape of a Staphylococcus aureus sequence type 45 strain, MCRF184
Source: BMC Genomics. 2019 Feb 8;20:123. doi: 10.1186/s12864-018-5394-2 (PMC6368776; doi:10.1186/s12864-018-5394-2)

Supplemental Figure 1A. Amino acid sequences of Phenol Soluble Modulins (PSMs) of *S. aureus* MCRF184

PSMα1	MGIIAGIIKVIKSLIEQFTGK	+1
PSMα2	MGIIAGIIKFIKGLIEKFTGK	+2
PSMα3	MEFVAKLFKFFKDLLGKFLGNN	+1
PSMα4	MAIVGTIIKIIKAIIDIFAK	+2
PSMβ1	MEGLFNAIKDTVTAAINNDGAKLGTSIVSIVENGVGLLGKLFGF	−2
PSMβ2	MTGLAEAIANTVQAAQQHDSVKLGTSIVDIVANGVGLIGKLFGF	−1

Supplemental Figure 1B. Genomic locations of Phenol Soluble Modulins (PSMs) of *S. aureus* MCRF184.

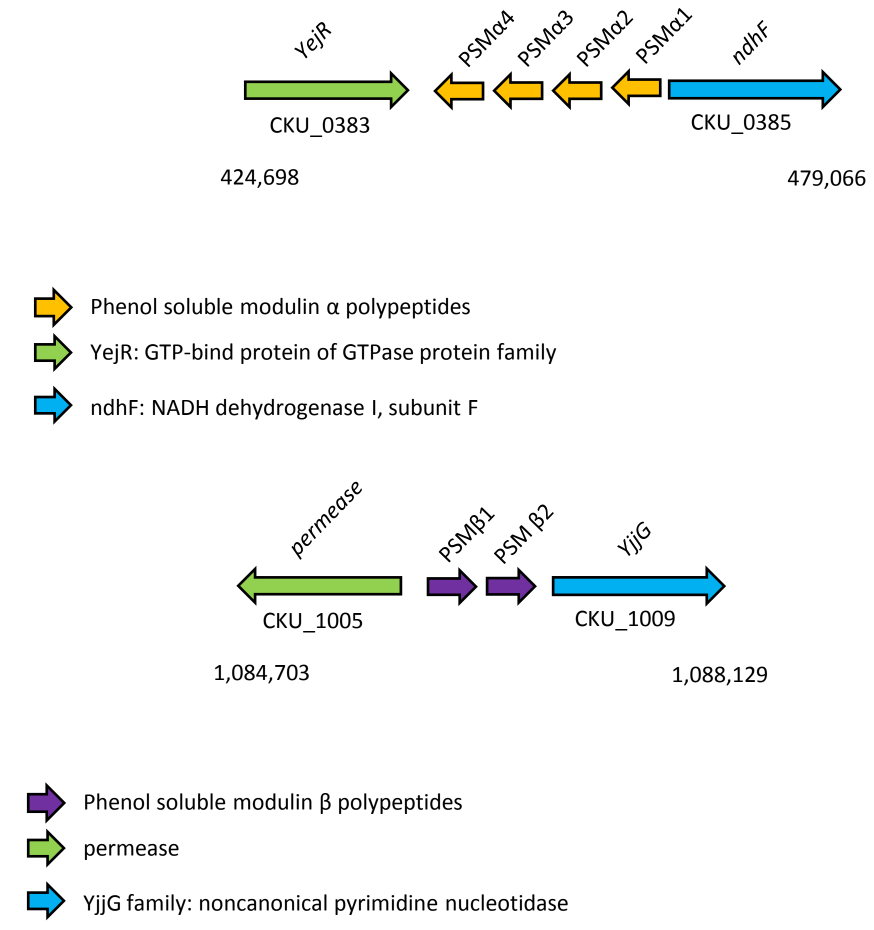

Supplemental Figure 1C. Predicted protein structures of Phenol Soluble Modulins (PSMs) of *S. aureus* MCRF184.

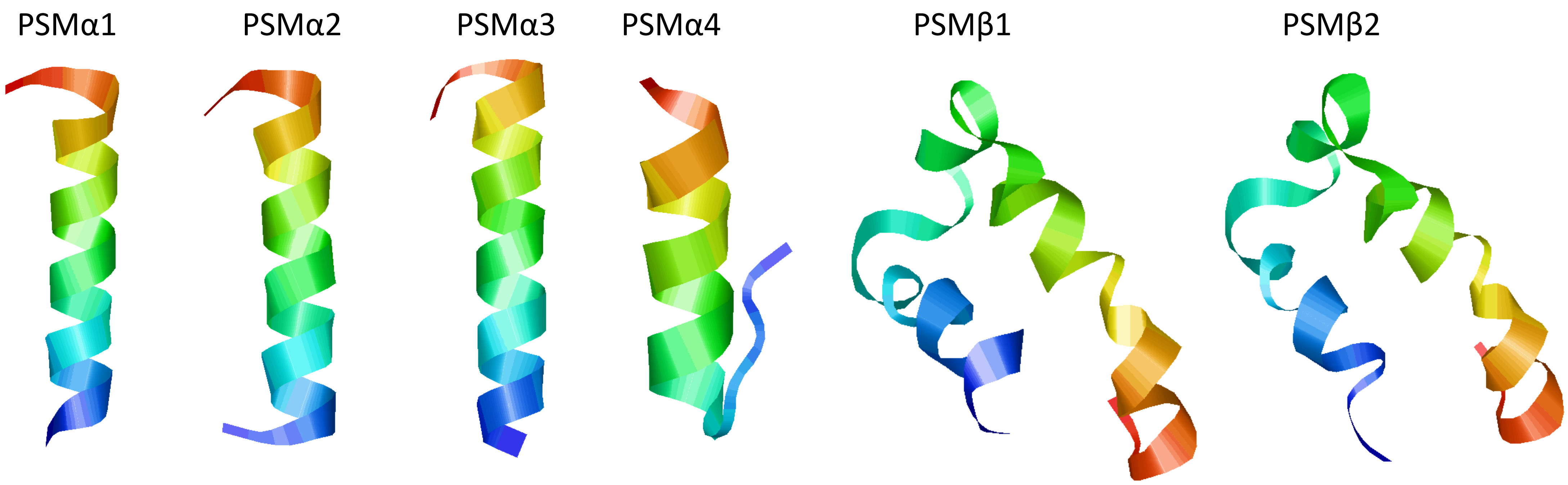

Supplement: Supplementary file 1 — Figure S1. Phenol soluble modulins (PSM) of S. aureus MCRF184. Panel 1A shows the amino acid sequences of the alpha and beta PSMs arranged from the N-terminus to the C-terminus. Numbers at the right show the net charge of the peptides at pH 7.0, rounded to whole numbers, and considering N-formylation of the initial methionine residue. The highlighted text identifies the amphipathic α-helical domain. Panel 1B shows the location of the genes coding for these PSMs in the genome of MCRF184 core genome. Panel 1C shows the predicted structure of the PSMS using PEP-FOLD (for the alpha PSMs) and SWISS-MODEL Workspace (for the beta PSMs). The residues are color-coded by their position in the peptide chain. Each chain is drawn as a smooth spectrum from blue through green, yellow and orange to red. The N-terminus of the peptides is colored red and the C terminuses are drawn in blue. The structures show the characteristic α-helical structure of the C-terminus ends of the PSMs. (PDF 334 kb) [file 12864_2018_5394_MOESM1_ESM.pdf]
